# Lower Serum Chloride at ICU Admission Is Associated With Acute Kidney Injury in Critically Ill Solid Tumour Patients: A Retrospective Cohort Study

**DOI:** 10.7759/cureus.95235

**Published:** 2025-10-23

**Authors:** Chenglian Wang, Longyong Li, Dinghua Liu, Yaoping Liu

**Affiliations:** 1 Intensive Care Medicine, Ganzhou Cancer Hospital, Ganzhou, CHN; 2 Orthopaedics and Interventional Medicine, Ganzhou Cancer Hospital, Ganzhou, CHN

**Keywords:** acute kidney injury, biomarker, cancer, chloride, critical care, prediction, solid tumours

## Abstract

Background

Acute kidney injury (AKI) is common in cancer intensive care units (ICUs) and is associated with high mortality. Although chloride plays a key role in renal haemodynamics, its clinical significance is often underemphasised. However, the relationship between serum chloride and AKI in oncological critical care remains unexplored in observational studies.

Methods

We analysed 846 consecutive solid tumour ICU admissions (≥3 days, 2018-2024) without AKI within 48 hours of admission. Hypochloraemia was defined as serum chloride <96 mmol/L. AKI was adjudicated using KDIGO creatinine criteria. Multivariable logistic regression and ROC analyses were performed.

Results

AKI developed in 416 patients (49.2%). Admission chloride was lower in those who developed AKI (97.0 ± 11.3 vs 104.0 ± 12.5 mmol/L, P < 0.001). After adjustment for illness severity (APACHE II score) and baseline renal function (eGFR), among other confounders, patients with hypochloraemia had 87% higher odds of AKI than those with normochloraemia (OR 1.87, 95% CI 1.14-2.60). Furthermore, each 1 SD decrement (11.5 mmol/L) in serum chloride raised the AKI risk by 46.5% (OR 1.465, 95% CI 1.134-1.792). Discriminative AUCs were 0.751 for any AKI and 0.819 for stage 3 AKI; optimal cut-offs (97 mmol/L and 92 mmol/L, respectively) lay at or below the reference range. Associations remained consistent across baseline estimated glomerular filtration rate strata.

Conclusions

Lower serum chloride is a readily identifiable and potentially modifiable predictor of AKI in critically ill solid tumour patients. The potential of real-time chloride monitoring and early repletion as low-cost AKI prevention strategies warrants prospective evaluation.

## Introduction

Acute kidney injury (AKI) is a global healthcare concern characterised by a rapid decline in kidney function, typically defined by a rapid increase in serum creatinine (Scr), a decrease in urine output, or both. This condition is closely linked to unfavourable clinical outcomes, heightened medical expenses, and increased morbidity and mortality [1,2].

Among patients with cancer, the incidence of AKI is disproportionately high; pooled estimates indicate that cancer-related AKI occurs in 12-66.5% of cases [3,4]. The wide range reflects substantial heterogeneity among cancer populations, with the highest incidence observed in patients with haematological malignancies undergoing intensive chemotherapy or stem cell transplantation, and those with advanced solid tumours experiencing sepsis or tumour lysis syndrome. The pathophysiology of AKI in these settings is multifactorial and often synergistic, arising from several key mechanisms. Contributors include tumour-related sequelae (for example, metabolic disturbances and infiltration), the nephrotoxic profile of many chemotherapeutic and targeted agents, and systemic complications such as sepsis. This complex landscape of renal injury, well documented in the literature [3-7], underscores the critical need to identify modifiable risk factors to mitigate AKI risk.

Management is correspondingly complex, requiring simultaneous attention to patient-related factors (adequate hydration and avoidance of nephrotoxins), cancer-specific issues (relief of urinary obstruction and correction of hypercalcaemia), and treatment-related variables (dose adjustment of chemotherapy and prompt recognition of ICI nephritis), ideally within a multidisciplinary framework [4]. Once admission to the intensive care unit (ICU) is required, renal replacement therapy (RRT) is frequently necessary and mortality rises steeply [7,8]. Identification of modifiable risk factors amenable to early intervention is therefore paramount.

Chloride is the predominant extracellular anion, crucial for maintaining plasma tonicity, electroneutrality, acid-base balance, and vascular tone [9,10]. Despite its physiological importance, it receives less clinical attention than sodium or potassium. Chloride disturbances are common in critically ill patients: hypochloraemia frequently arises from gastrointestinal losses, diuretics, or dilutional states, while hyperchloraemia is often associated with fluid loss or metabolic acidosis [9,10]. This underappreciation of chloride’s clinical significance [11] makes it a compelling candidate for further investigation in the context of cancer-related AKI.

Recent evidence indicates that dyschloraemia carries independent prognostic weight across diverse settings, including liver disease [12], cardiovascular events [11-13], kidney disease progression, and all-cause mortality [14]. Notably, in individuals with baseline normal renal function, lower serum chloride has been associated with a higher risk of contrast-associated AKI [15], while among critically ill patients with established AKI, admission hypochloraemia amplifies the probability of in-hospital death [14]. Furthermore, 2023 population-based data from the National Health and Nutrition Examination Survey demonstrate that decreased serum chloride concentrations independently predict both overall and cancer-specific mortality in adults [16]. Whether hypochloraemia predisposes critically ill oncology patients to AKI remains unexplored.

Therefore, this study primarily aimed to test the hypothesis that hypochloraemia at ICU admission is independently associated with an increased risk of incident AKI in critically ill solid tumour patients. Secondarily, we sought to determine optimal serum chloride thresholds for AKI prediction and to evaluate their discriminative performance.

## Materials and methods

Study design

We conducted a single-centre, retrospective cohort study in the 20-bed oncological intensive care unit (ICU) of Ganzhou Cancer Hospital, a 710-bed tertiary cancer centre in Jiangxi Province, China, from January 2018 to December 2024. The institutional ethics committee approved the study protocol (No. 2025-233) and waived informed consent because of the observational design. Patient confidentiality was protected in accordance with institutional regulations and the ethical principles of the Declaration of Helsinki.

Study population and data collection

Consecutive adults with histologically confirmed solid tumours who were admitted to the ICU for ≥3 days were screened. The minimum three-day ICU stay was required to ensure sufficient follow-up for the development of hospital-acquired AKI and to guarantee the availability of serial serum creatinine measurements necessary for reliable AKI adjudication. This duration was selected based on a pre-study analysis (data not shown) to optimally balance adequate observation with data completeness. Patients were eligible if they were (1) aged between 18 and 80 years, (2) without evidence of AKI at ICU admission or within the first 48 hours of ICU stay, and (3) had baseline serum chloride records at ICU admission, as well as serial serum creatinine values and clinical information available. Exclusion criteria were haematological malignancy, any condition precluding reliable AKI assessment, or missing data required to calculate illness-severity scores.

We retrieved patients’ data from the local database and electronic medical records. The following demographic and clinical data were collected: cancer type (local/regional or metastatic), site of solid cancer, Charlson Comorbidity Index (CCI), and Acute Physiology and Chronic Health Evaluation (APACHE) II score at ICU admission. Serum chloride levels were measured in the hospital’s central laboratory using ion-selective electrode methodology on a Roche Cobas c501 analyser, as per the standard institutional protocol.

Definitions and outcomes

The primary endpoint was the occurrence of AKI during the ICU stay. AKI was identified and staged according to the Kidney Disease: Improving Global Outcomes (KDIGO) serum creatinine criteria: an absolute increase ≥ 0.3 mg/dL within 48 hours, or a ≥ 1.5-fold increase from the known or presumed baseline value within the previous seven days [17]. Owing to the retrospective design, hourly urine output data were incomplete; therefore, the KDIGO urine-output criteria were not applied. Stages 2 and 3 were combined as "advanced AKI," with stage 3 alone considered "severe AKI."

Baseline laboratory values were defined as the first measurement obtained within 24 hours of ICU admission, except for Scr, whose lowest value recorded within the three months preceding ICU admission was taken as baseline. This baseline Scr was used to calculate the estimated glomerular filtration rate (eGFR) using the Chronic Kidney Disease Epidemiology Collaboration equation [18], on the basis of which patients were categorised into eGFR stages.

Hypochloraemia was defined as a serum chloride concentration < 96 mmol/L and hyperchloraemia as ≥ 106 mmol/L, corresponding to the lower and upper bounds of the institutional reference interval (96-106 mmol/L) [15].

The CCI was used to quantify baseline comorbid burden. Illness severity on ICU admission was assessed with the APACHE II score.

Statistical analysis

Continuous variables are reported as mean ± SD or median (Q25, Q75), based on the assessment of data distribution using the Shapiro-Wilk test. Categorical variables are expressed as number (%). One-way analysis of variance (ANOVA) was used to assess differences among groups for normally distributed continuous variables. When the overall ANOVA indicated significant differences, post hoc pairwise comparisons were conducted using the Bonferroni correction to adjust for multiple testing. For continuous variables that did not follow a normal distribution, the Kruskal-Wallis H test was used to compare differences among groups. If the Kruskal-Wallis test indicated significant differences, pairwise comparisons were performed using Dunn’s test with Bonferroni correction for multiple comparisons. The χ² test was used for categorical variables. Multivariate logistic regression was employed to identify independent associations between electrolyte parameters and incident AKI. Covariates with P < 0.10 on univariate analysis were entered into the multivariate model, provided the variance inflation factor was < 10. Receiver-operating characteristic (ROC) curves were generated to determine optimal chloride thresholds that maximised the Youden index. Missing data were handled by complete-case analysis. The proportion of missing data for any variable used in the models was minimal (< 5%). Given the low rate of missingness and the absence of a systematic pattern (as assessed visually and by comparing baseline characteristics between cases with and without missing data), it is unlikely to have introduced substantial bias into the results.

All tests were two-tailed, and P < 0.05 was considered statistically significant. Analyses were performed with IBM SPSS Statistics for Windows, Version 26 (Released 2019; IBM Corp., Armonk, New York).

## Results

Baseline characteristics

**Figure 1:**
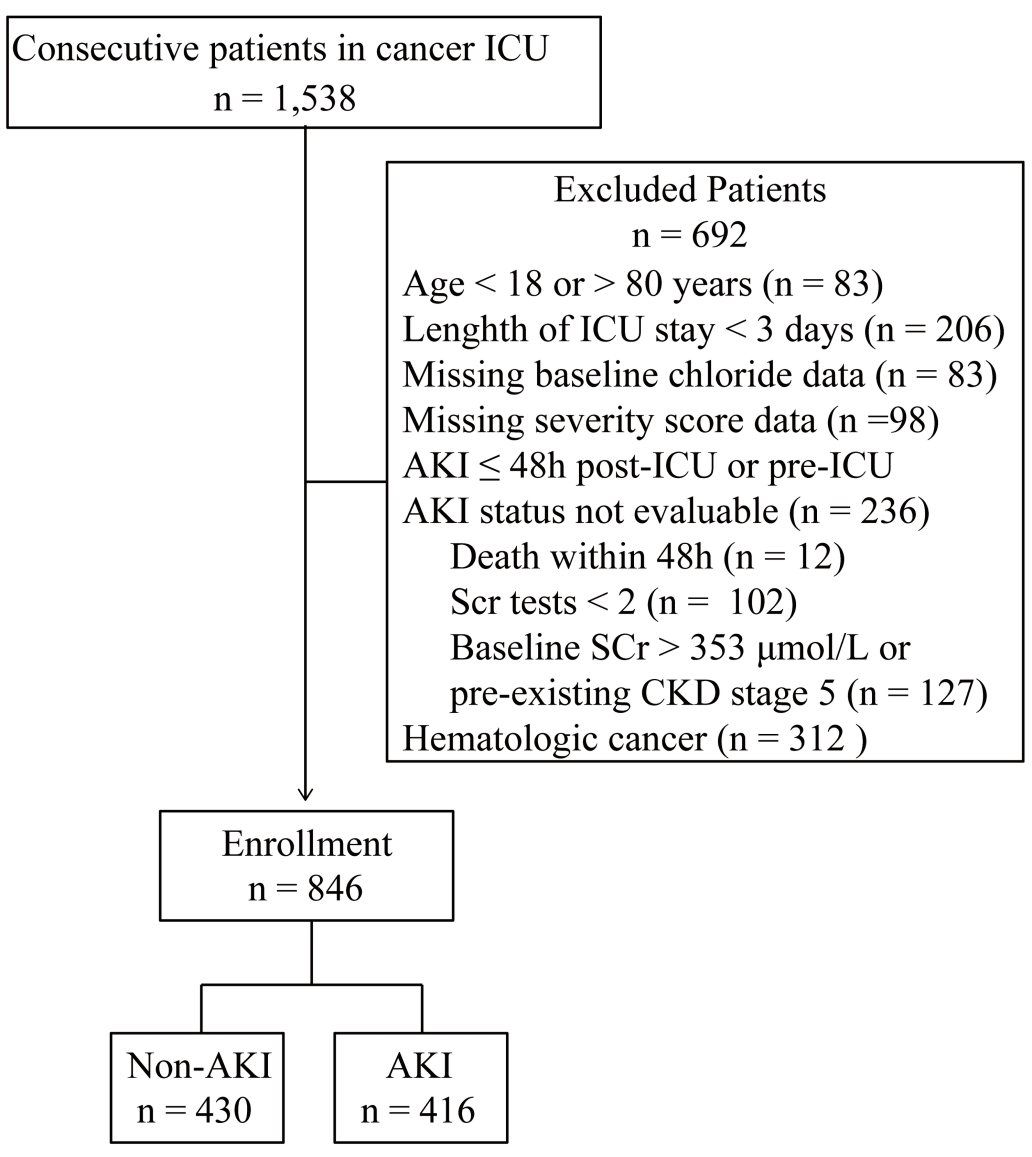
Inclusion and exclusion criteria. ICU: intensive care unit; AKI: acute kidney injury; Scr: serum creatinine; CKD: chronic kidney disease.

After screening 1,538 consecutive admissions, 846 adult solid tumour patients (median age, 63 years; 50.4% male) who met all inclusion criteria were retained (Figure 1). AKI developed in 416 patients (49.2%) during their ICU stay. Patients who developed AKI were older and had more frequent metastatic disease, heart failure, and gastrointestinal primaries, higher CCI and APACHE II scores, lower haemoglobin and serum albumin levels, and higher BUN (all P < 0.05; Table 1).

**Table 1:** Clinical characteristics. SD: standard deviation; AKI: acute kidney injury; CCI: Charlson Comorbidity Index; APACHE II: Acute Physiology and Chronic Health Evaluation II; WBC: white blood cell; eGFR: estimated glomerular filtration rate.

Variables	Total (n = 846)	Non-AKI (n = 430)	AKI (n = 416)	P Value
Age, years (mean (SD))	61.3±18.1	53.8±15.4	68.7±20.3	<0.001
Male, n (%)	416 (50.4)	209 (48.6)	206 (49.5)	0.790
Metastatic cancer, n (%)	502 (59.3)	184 (42.8)	318 (76.4)	<0.001
Site of cancer, n (%)				0.021
Gastrointestinal	221 (26.12)	72 (19.94)	149 (30.72)	
Hepatobiliary	79 (9.34)	34 (9.42)	45 (9.28)	
Head and neck	30 (3.55)	18 (4.99)	12 (2.47)	
Lung	324 (38.30)	142 (39.34)	182 (37.53)	
Breast	109 (12.88)	58 (16.07)	51 (10.52)	
Urological	44 (5.20)	16 (4.43)	28 (5.78)	
Other	39 (4.61)	21 (5.82)	18 (3.71)	
Comorbid disease, n (%)				
Diabetes	316 (37.35)	88 (28.30)	228 (42.62)	0.415
Hypertension	340 (40.19)	162 (37.33)	178 (43.2)	0.072
Coronary artery disease	181 (21.39)	89 (20.32)	92 (22.44)	0.452
Heart failure	310 (36.64)	112 (31.02)	198 (40.82)	0.003
CCI (median (Q_25_, Q_75_))	8 (3,12)	5 (3,7)	10 (5,13)	<0.001
APACHE Ⅱ (median (Q_25_, Q_75_))	11 (6,13)	8 (6,10)	11 (7,14)	<0.001
Laboratory data (mean (SD))				
WBC, 10^9^/L	8.7±3.6	6.8±2.4	10.8±4.9	<0.001
Haemoglobin level, g/L	110.9±23.7	118.1±24.3	102.3±22.3	<0.001
Serum albumin, g/L	35.1±8.0	37.2±9.2	33.2±7.5	<0.001
Blood urea nitrogen, mmol/L	11.6±3.9	10.8±3.4	12.5±4.7	<0.001
eGFR, ml/min/1.73m^2^	74.7±24.5	79.4±23.8	68.3±21.4	<0.001

Serum chloride profile

**Figure 2:**
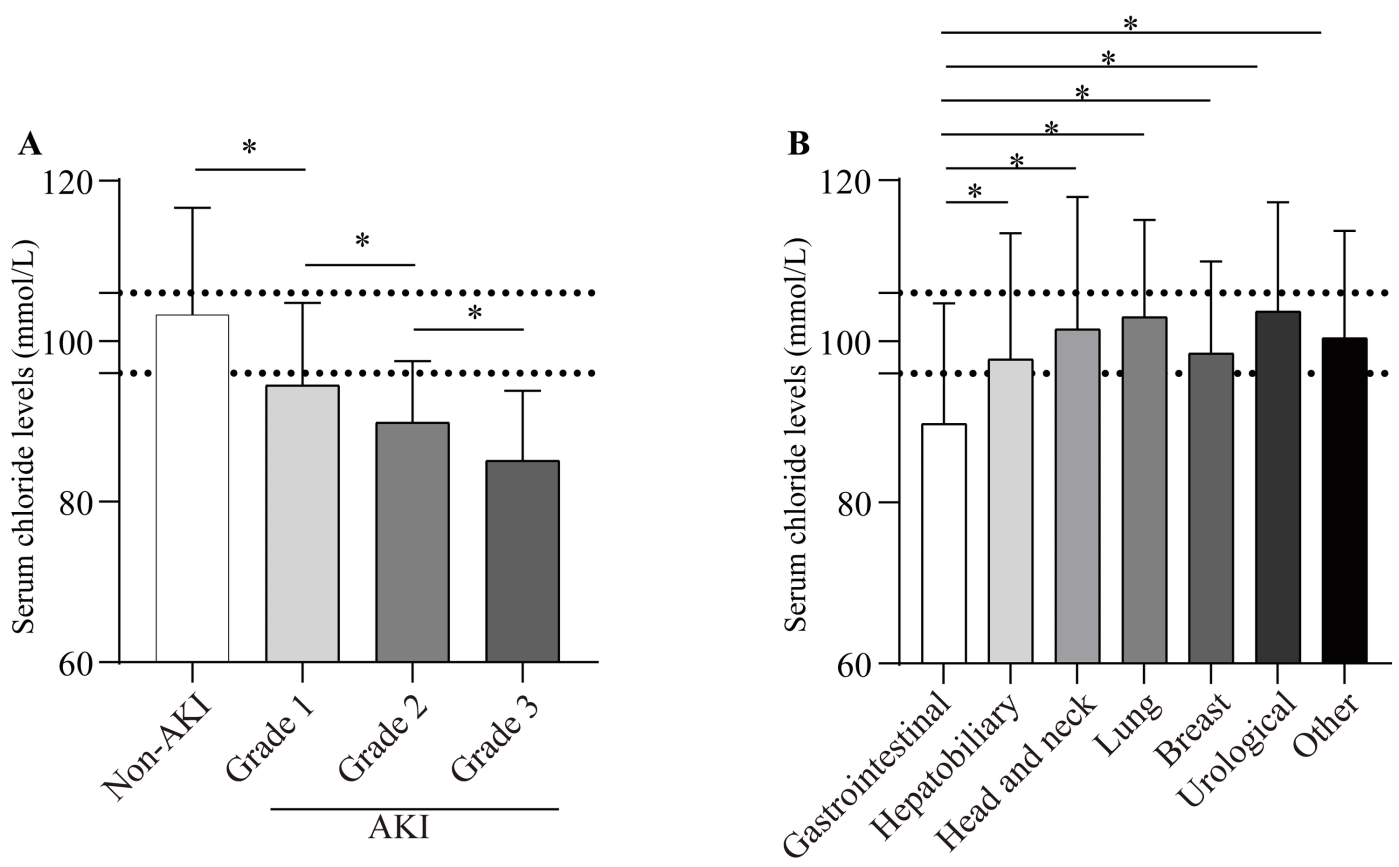
Serum chloride distributions according to AKI status and tumour type. Panel A illustrates that baseline chloride levels were significantly lower in patients who developed acute kidney injury (AKI). Chloride levels decreased progressively with increasing severity of AKI. Panel B shows that, among the major malignancies, patients with gastrointestinal tumours exhibited the lowest chloride values. *P < 0.05 indicates a significant difference compared with other tumour types by post hoc analysis. The dashed horizontal line denotes the institutional reference range (96-106 mmol/L).

Of the total cohort, 403 patients (47.6%) met the criteria for hypochloraemia, while 214 patients (25.3%) met the criteria for hyperchloraemia. Chloride concentrations were significantly lower in patients who subsequently developed acute kidney injury (AKI) (97.0 ± 11.3 mmol/L) compared with those who did not (104.0 ± 12.5 mmol/L; P < 0.001). Moreover, chloride levels decreased in a stepwise manner across higher AKI stages (Figure 2A). Among different cancer types, patients with gastrointestinal malignancies had a mean chloride level of 89.8 ± 14.9 mmol/L, which was significantly lower than that observed in other tumour types (all P values < 0.05; Figure 2B).

Association between chloride metrics and incident AKI

After multivariable adjustment for age, metastatic status, CCI and APACHE II scores, diabetes, heart failure, peripheral white blood cell count, albumin, and haemoglobin, hypochloraemia remained independently associated with an increased risk of AKI (OR 1.87, 95% CI 1.14-2.60; P = 0.013) compared with the normochloraemia stratum. In contrast, hyperchloraemia was not significantly associated with AKI risk (OR 0.96, 95% CI 0.90-1.11; P = 0.35).

To further quantify the chloride-AKI association in clinically meaningful terms, baseline chloride was modelled per one standard deviation (SD) decrement (11.5 mmol/L). Each 1 SD decrease in chloride was associated with a 46.5% increase in the odds of AKI (OR 1.465, 95% CI 1.134-1.792; P = 0.022) (Table 2).

**Table 2:** Acute kidney injury-associated risk factors. OR: odds ratio; CCI: Charlson Comorbidity Index; APACHE II: Acute Physiology and Chronic Health Evaluation II; WBC: white blood cell; eGFR: estimated glomerular filtration rate.

Variables	OR	95% CI	P Value
Age, per 5-year increase	1.235	1.114–1.347	<0.001
Metastatic cancer	1.419	1.108–1.735	<0.001
Site of cancer			
Non-gastrointestinal	Reference		
Gastrointestinal	2.019	1.148–2.937	<0.001
Comorbid diabetes	2.134	1.723–2.566	<0.001
Comorbid hypertension	1.039	0.824–1.206	0.189
Comorbid heart failure	3.298	1.938–4.172	<0.001
CCI, per 1-point increase	1.872	1.176–2.504	<0.001
APACHE Ⅱ, per 1-point increase	3.079	1.895–4.012	<0.001
WBC, per 1×10^9^/L increase	1.107	0.903–1.375	0.215
Haemoglobin level, per 10 g/L decrease	1.205	0.982–1.417	0.089
Serum albumin, per 5 g/L decrease	1.208	1.083–1.415	<0.001
eGFR, per 15 ml/min/1.73m^2^ decrease	1.439	1.143–2.092	<0.001
Serum chloride, per 11.5 mmol/L decrease	1.465	1.134–1.792	<0.001

To examine whether the association between hypochloraemia and AKI varied with baseline kidney function, we conducted multivariable logistic regression analyses within eGFR-stratified subgroups. The findings indicated that the relationship between reduced serum chloride and AKI risk remained consistent across all strata (Table 3).

**Table 3:** Reduced blood chloride levels and AKI risk across different eGFR strata. eGFR: estimated glomerular filtration rate; AKI: acute kidney injury.

eGFR stratum (mL/min/1.73m^2^)	n	Any AKI		AKI stage 2–3		AKI stage 3	
		OR (95% CI)	P Value	OR (95% CI)	P Value	OR (95% CI)	P Value
15–29	72	1.382 (1.029–1.793)	0.037	1.989 (1.347–2.314)	<0.001	2.405 (1.378–3.235)	<0.001
30–45	145	1.573 (1.187–1.904)	<0.001	1.298 (0.984–1.405)	0.054	1.944 (1.278–2.630)	<0.001
45–59	307	1.489 (1.131–1.753)	<0.001	1.649 (1.147–2.136)	<0.001	1.685 (1.204–2.073)	<0.001
≥60	322	1.633 (1.194–2.022)	<0.001	1.675 (1.233–1.908)	<0.001	1.544 (1.198–1.939)	<0.001

Discriminative performance of serum chloride

ROC analysis indicated that baseline serum chloride discriminated any AKI (AUC 0.751), stage 2-3 AKI (0.768), and stage 3 AKI (0.819) with moderate-to-excellent accuracy, with the AUC increasing stepwise with AKI severity (Figure 3). The optimal cut-offs for any AKI and stage 2-3 AKI (97.3 and 97.4 mmol/L) sat just below the lower limit of the clinical reference range (96-108 mmol/L). These thresholds lie within or marginally below the routine reference range, implying that even “low-normal” chloride may be an actionable warning sign worthy of integration into early AKI alerts. The threshold for stage 3 AKI (91.8 mmol/L) fell markedly below this range, underscoring the heightened risk conferred by overtly low chloride levels (Table 4).

**Table 4:** Discriminative performance of serum chloride for AKI. AKI: acute kidney injury; AUC: area under the curve.

Endpoints	AUC (95% CI)	Cut-off (mmol/L)	Sensitivity (%)	Specificity (%)	P value
Any AKI	0.751 (0.718–0.783)	97.3	71.9	68.8	< 0.001
AKI stage 2–3	0.768 (0.735–0.802)	97.4	87.2	58.4	< 0.001
AKI stage 3	0.819 (0.748–0.874)	91.8	82.1	67.4	< 0.001

## Discussion

In this retrospective cohort study of 846 critically ill patients with solid tumours, we found that hypochloraemia at ICU admission was independently associated with incident AKI, nearly doubling the risk (OR 1.87). It is important to note the limitations of this study, including its retrospective, single-centre design and the use of a single static measurement of serum chloride at admission, which precludes assessment of dynamic chloride trajectories. Nonetheless, the risk increased in a stepwise manner across AKI stages. This association remained robust across all eGFR strata, suggesting that chloride depletion may act as a modifiable renal vulnerability factor, even in patients without overt baseline CKD. Notably, the discriminative performance of serum chloride for severe AKI (AUC 0.819) rivalled that of established illness-severity scores, implying incremental value for early risk stratification in oncological ICU settings.

Chloride is the principal extracellular anion that modulates tubuloglomerular feedback and afferent arteriolar tone [19]. When chloride levels fall, this feedback is attenuated: afferent arterioles dilate, systemic hypotension is transmitted to the glomerulus, and GFR declines, independent of circulating volume [19-21]. Simultaneously, reduced tubular chloride disinhibits renin release, amplifying Ang II-mediated efferent arteriolar constriction and exacerbating glomerular ischaemia [22,23]. This impairment of renal autoregulation renders the kidney more susceptible to haemodynamic insults. Critically ill solid tumour patients are particularly vulnerable to this cascade. Our findings align with this pathophysiology. Notably, patients with gastrointestinal primaries had both the lowest serum chloride levels among major tumour types and a higher incidence of AKI (Table 1 and Figure 2B), a combination that may partly explain their elevated AKI risk. A substantial proportion of these patients, especially those with gastrointestinal malignancies, experience profound chloride-rich fluid losses from vomiting, diarrhoea, or drainage, which can lead to hypochloraemia even before overt sodium depletion or hypovolaemia occurs. This state of chloride depletion, often manifesting as hypochloraemic metabolic alkalosis, creates a background of compromised renal reserve. When superimposed on other common nephrotoxic insults in this population, such as sepsis, nephrotoxic drugs, or contrast media, the kidney’s ability to compensate is overwhelmed, thereby precipitating AKI. These chloride-related pathophysiological mechanisms may explain why even low-normal chloride levels emerged as a significant predictor of AKI.

Previous investigations of the chloride-AKI association in mixed critically ill cohorts have yielded conflicting results, largely owing to heterogeneous case mix and divergent chloride metrics. In a retrospective single-centre study of 1,221 unselected ICU patients, baseline chloride did not differ between those who developed AKI and those who did not, yet the peak and mean chloride values were 4.1 mmol/L and 0.9 mmol/L higher, respectively, in the AKI group (both P < 0.05) and remained independently predictive after multivariable adjustment [24]. Conversely, in a septic ICU population where 29% presented with hyperchloraemia (≥ 110 mmol/L), neither baseline hyperchloraemia nor the magnitude of chloride increase predicted AKI or moderate-to-severe AKI following correction for confounders [25]. In our study, although about one quarter of the cohort exhibited hyperchloraemia on ICU admission, logistic modelling revealed no graded relationship between higher chloride strata and incident AKI. This discrepancy likely reflects both the exclusive enrolment of critically ill cancer patients and the fact that our primary objective was to test admission chloride as a static risk marker for subsequent AKI. Consequently, we did not track post-admission chloride trajectories, which could have risen after AKI had already started.

The identified chloride thresholds (97.3 mmol/L for any AKI and 91.8 mmol/L for stage 3 AKI) approximate or fall below the conventional reference limit, indicating that early chloride monitoring could enhance AKI-prediction models that are presently predicated predominantly on creatinine. Embedding real-time chloride alerts within electronic health records, comparable to the “nephrotoxic-exposure dashboards” already deployed for AKI alerts, may facilitate timely interventions [26]. By directing attention to the lower physiological range, our data establish hypochloraemia as an independent and potentially modifiable renal risk factor in critically ill cancer patients who concurrently sustain multiple nephrotoxic insults.

Study strengths include the homogeneous oncological ICU population, rigorous KDIGO adjudication of AKI, and comprehensive adjustment for cancer- or ICU-specific confounders (metastatic burden, CCI, APACHE II). Limitations are inherent to the retrospective, single-centre design: unmeasured residual confounding (for example, detailed daily fluid balance, exposure to nephrotoxic agents or specific chemotherapeutic regimens, and cancer-related metabolic disturbances) cannot be excluded, and their potential role, while discussed, was not quantitatively assessed in our models. Although serum chloride was measured using a standardised ion-selective electrode assay in a central laboratory, we lacked data on its reproducibility over time for individual patients. Serial chloride trajectories post-admission were not analysed, precluding assessment of chloride variability as a dynamic risk modifier. Urine-output criteria were not utilised, potentially underestimating AKI incidence; however, this bias is likely non-differential with respect to chloride strata. Finally, external validity to haematological malignancies or non-ICU oncology settings awaits confirmation.

Prospective multicentre cohorts should validate our chloride thresholds across diverse oncological populations and explore chloride trajectory patterns (slope, nadir, time-weighted average) using high-granularity electronic data, with careful adjustment for dynamic clinical variables such as fluid balance and diuretic use. Mechanistic studies pairing renal tissue transcriptomics with chloride manipulation in murine cancer models could clarify whether chloride repletion attenuates tubular apoptosis or macrophage-driven inflammation. Interventional pilot trials randomising hypochloraemic patients to chloride repletion versus standard care are warranted to establish causality and inform guideline-level recommendations.

## Conclusions

Hypochloraemia independently heightens AKI risk in oncological ICU patients. Vigilant monitoring and early correction of hypochloraemia may represent a potential simple, cost-effective strategy to reduce AKI risk in critically ill cancer patients.
